# Compliance with Headgear Evaluated by Force- and Temperature-Sensitive Monitoring Device: A Case-Control Study

**DOI:** 10.3390/bioengineering11080789

**Published:** 2024-08-05

**Authors:** Francesca Cremonini, Ariyan Karami Shabankare, Daniela Guiducci, Luca Lombardo

**Affiliations:** Postgraduate School of Orthodontics, University of Ferrara, Via Luigi Borsari 46, 44121 Ferrara, Italy

**Keywords:** headgear, compliance, Smartgear

## Abstract

The aim was to objectively assess compliance in patients prescribed headgear and evaluate the impact of monitoring awareness, treatment duration, gender, and age on compliance levels. A total of 22 patients with Class II malocclusion wore the headgear integrated with the force and temperature sensitive Smartgear monitoring system (Smartgear, Swissorthodontics AG, Cham, Switzerland). Patients were instructed to wear the headgear for 13 h daily over a 3-month period. Randomly, 11 patients were informed that they monitored and 11 were not informed. Data were organized using Microsoft Excel and analyzed using R for statistical estimates, graphs, and hypothesis testing. Smartgear recorded an average daily compliance of 6.7 h. No statistically significant differences were found in cooperation between study group and control group over the 3 months of treatment, regardless of gender and age. However, there was slight greater cooperation in the first month than in the other months, and patients ≤10 years of age had almost 2 h more cooperation than their older counterparts. Moreover, the informed group exhibited an average of 1.1 more hours of cooperation per day than the uninformed group, which may carry clinical significance. This cooperation primarily occurred at night and was found to be statistically significant. Compliance among young patients typically remained lower than the prescribed level, regardless of their gender and psychological maturity. Although an awareness of monitoring does not seem to improve compliance, implementing such systems could still offer dentists a valuable means of obtaining objective information about their patients’ adherence.

## 1. Introduction

For several decades, the application of extraoral traction to the maxilla has been a common practice to restrain or redirect growth in Class II patients, particularly those with maxillary excess [1]. This inhibitory effect on maxillary anterior displacement has primarily been achieved using the headgear appliance, which can be categorized into three types based on the direction of applied force: high-pull headgear (anchored at the upper back of the head), cervical headgear (anchored at the back of the neck), and combi-headgear (anchored at both sites).

Clinical studies have shown that the outcomes of headgear treatment are attributable partly to dental changes in the sagittal and vertical planes and partly to skeletal changes. Headgear treatment has been found to effectively alter maxillary growth both sagittally and vertically, with reported instances of palatal plane rotation and changes in anterior face height too [2]. Many authors emphasize the type of headgear, the magnitude of applied force, and the direction of pull as significant factors influencing the modification of effects to be considered [3]. Drawing from high-quality evidence, headgear treatment demonstrates a short-term reduction in the SNA angle, which remains unaffected by confounding influences on the subspinale point and correlates with the severity of the initial SNA angle discrepancy [4]. Consequently, headgear emerges as a promising and efficient treatment avenue for managing Class II malocclusion with maxillary prognathism [5]. Additionally, based on moderate-quality evidence, headgear treatment may mitigate the risk of dental trauma over subsequent years, underscoring the importance of considering this aspect in the treatment planning for Class II patients at high risk of dental injury [6]. However, patient compliance plays a crucial role in ensuring that the headgear is sufficiently effective. Clinicians often face a persistent challenge when it comes to objectively confirming whether patients are actually using the headgear as instructed. As a result, various methods and tools have been proposed over the years to improve the understanding of patient compliance in this context. One potential approach involves tracking the daily time that patients wear their headgear, which can provide clinicians with valuable insights into treatment progress and serve as a motivational tool to encourage patient cooperation.

Various methods have been employed to evaluate how well patients adhere to the prescribed use of headgear. These methods rely on established criteria, including (1) molar mobility, (2) the cleanliness of extra-oral traction components and headgear straps, (3) speed and ease in wearing the appliance, (4) interdental spacing changes, (5) molar alignment relative to pre-treatment references or cephalometric analysis, and (6) the retention of orthodontic anchorage. However, it is worth noting that the subjective nature of these measurement tools poses a significant challenge in accurately predicting and quantifying patient cooperation [3,7]. Some have turned to methods like questionnaires or calendars, which patients complete to track the hours of device use; however, it should be kept in mind that it has been widely shown in the literature that patients tend to overestimate how much they use compliance-dependent equipment [8,9,10]. Some authors have used stopwatches instead; however, Banks and Read reported that only 4 of 13 timing devices produced average accuracy values above 90% [11].

Objective methods are currently available to assess the patient’s level of cooperation in wearing headgear. In particular, reference is made to modern temperature-sensitive sensors, such as the TheraMon (TheraMon, MC Technology GmbH, Hargelsberg, Austria o Dentaurum Italia Spa, Bologna, Italy) or Smart Retainer (Smart Retainer, Scientific Compliance, Atlanta, GA, USA). These devices can detect whether the orthodontic appliance is inserted inside the oral cavity, as they detect a temperature similar to that of the body (between 35° and 37 °C). Due to their small size, both devices can integrate seamlessly with standard removable orthodontic appliances [11,12,13]. However, more specific in assessing compliance, since it can detect not only temperature but also applied force, is the Smartgear compliance monitoring device (Smartgear, Swissorthodontics AG, Cham, Switzerland), designed specifically for monitoring the time of headgear use. For these reasons, this objective compliance monitoring system was chosen among several in the market. The detection of both temperature and force makes it possible to minimize the possibility of error, so the device is able to record more accurately when the headgear is put on and when it is removed.

The existing literature recognizes that monitoring the use of headgear can improve collaboration, but there is a shortage of comprehensive research quantifying this effect. This study aimed to accurately assess patient collaboration with headgear using objective assessment tools and to gain a detailed understanding of how the awareness of being monitored can influence device usage.

## 2. Materials and Methods

A total of 22 patients (13 boys and 9 girls) aged between 7 and 18 years (mean age of 11.1 years) treated at the Ferrara University Orthodontic Clinic formed the initial sample. The observation period was 3 months. All patients were treated with an initial phase of skeletal maxillary expansion, followed by headgear appliance for the correction of Class II skeletal malocclusion. All clinical procedures were performed by a single operator. The study design was reviewed and approved by the Ethics Committee of the Ferrara University Postgraduate School of Orthodontics (Via Luigi Borsari 46, Ferrara, Italy; approval number 6/2022).

**Figure 1:**
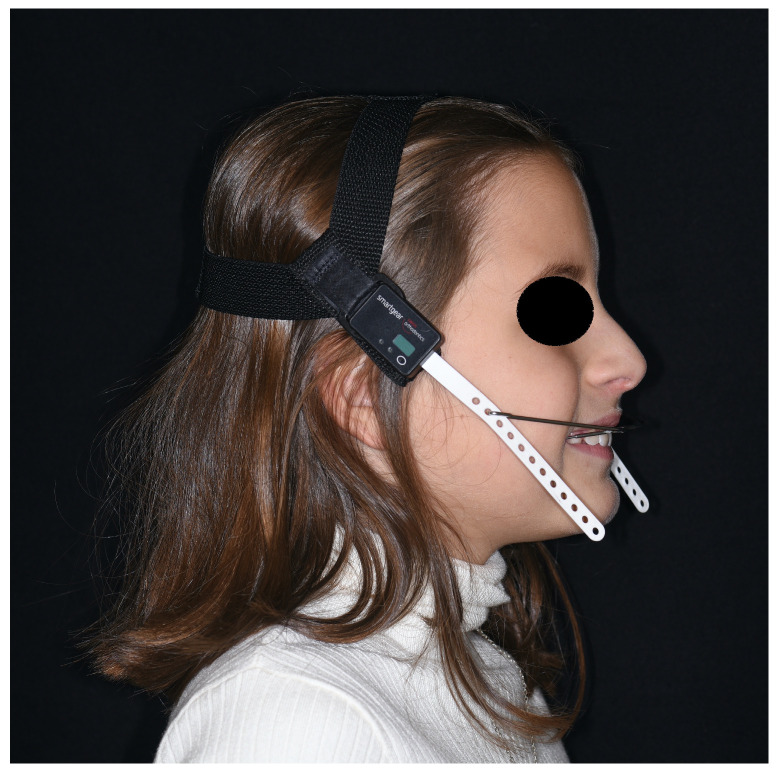
Smartgear compliance monitoring device integrated in headgear.

**Figure 2:**
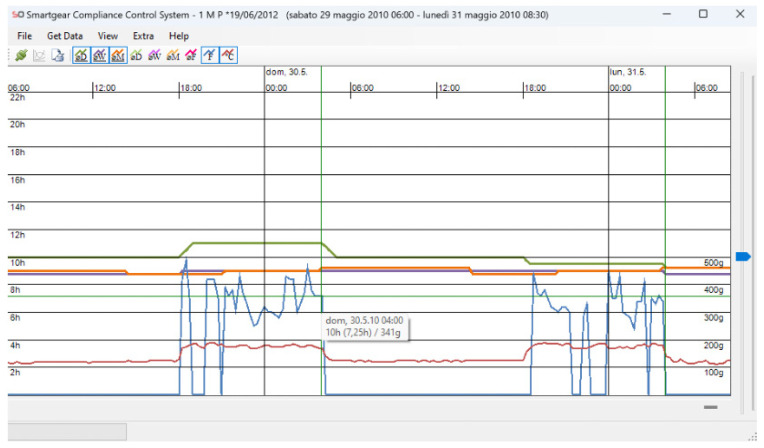
Example of a graph generated by the software.

All headgears, before being given to the patients, were equipped with a temperature and force-sensitive module (that can be integrated both into the cervical strap and the cranial cap of the extraoral traction) that measures temperature and activated force intensity once a minute, and the average values are calculated every 15 min (Figure 1). The accuracy of temperature and force measurements is 1 °C and 10 g, respectively. The module also allows the time and date of the headgear insertion/removal to be recorded. The information obtained can be transferred via an infrared system to the computer of the clinician, read via the Smartgear Compliance Control System software (version 2.1.2, Swissorthodontics AG, Cham, Switzerland), and visualized via graphs, which subsequently can be converted to table format for easier data analysis (Figure 2).

Each patient was given their headgear, and based on the specific case’s needs, it was either high-pull or low-pull traction. They were then instructed to wear it for a total of 13 h per day. A total of 11 randomly selected patients in a 1:1 ratio were informed that their appliance had been fitted with a sensor to accurately monitor their compliance (indicated as study group), whereas 11 were kept blind to this fact for the duration of their treatment (indicated as control group).

At each monthly check-up, the operator ensured that the appliance exerted a 500 g force on each side, and if the traction was not adequate, they would increase the insertion hole of the external arch by one or more to restore the appropriate force. Also, the data recorded by the sensor were downloaded onto the software Smartgear Compliance Control System, and the redout graphs were generated. Patients who were aware of the monitoring process were presented with their usage data and, subsequently, encouraged to enhance their compliance. In contrast, patients unaware of the monitoring were not provided with their data but instead received motivational guidance based on their observed clinical progress.

At the end of the treatment observation period (3 months), the recorded values were exported from the software into an Excel spreadsheet (Microsoft Excel 2015; Microsoft Corp., Redmond, WA, USA). They were then statistically analyzed by a single statistician using Student’s *t*-test (*p* < 0.05), the Wilcoxon–Mann–Whitney test (*p* < 0.05), ANOVA (*p* < 0.05), and the Kruskal–Wallis test (*p* < 0.05). Parameters considered were the mean compliance of the overall sample and differences ascribable to observation interval, gender, age, and an awareness of being monitored. 

The headgear was considered used when force was above zero (and temperature equal or greater of 23 °C).

## 3. Results

**Figure 3:**
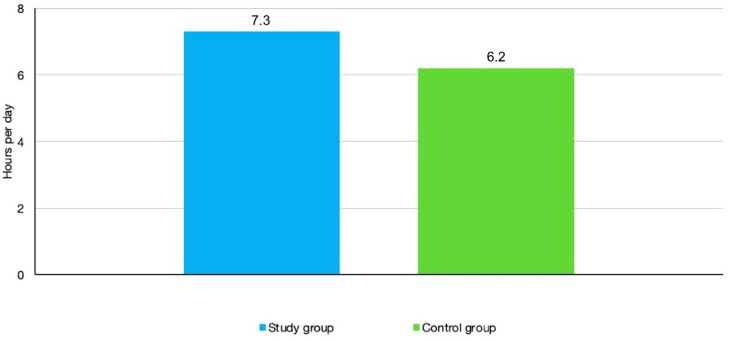
Compliance in study and control groups.

When prescribed 13 h a day, the mean compliance time revealed in the sample population of all patients was 6.7 h per day, that is, 46.15% of what was required. Although mean compliance was higher in the study group (those who were informed about the monitoring) than with the control group (those who were not informed about the monitoring), specifically 7.3 h per day compared to 6.2 h per day, the difference was not found to be statically significant (Figure 3).

**Figure 4:**
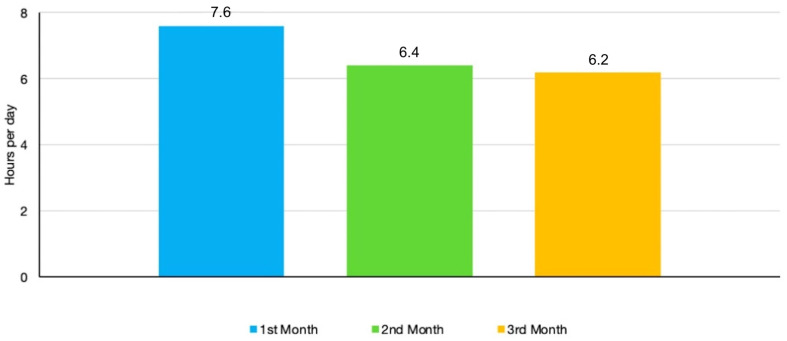
Compliance trend during the 3 months of observation.

The mean compliance remained largely stable during the 3 months of observation after a slight rise from the first (7.6 h/day) to the second month (6.4 h/day) and third month (6.2 h/day). No statistically significant differences were found between the monthly figures (Figure 4). In Table 1, the differences in daily compliance, measured in hours of device usage, between the two groups over the 3-month observation period can be seen, while Table 2 shows the average daily compliance of each individual patient over the same period.

Likewise, there was no statistically significant difference between genders, although boys did tend to be slightly more compliant than girls (respectively, 7.1 and 6.2 h per day).

When the sample was subdivided by age, however, it became evident that compliance decreased with increasing age: compliance in the group of patients 10 years old or younger and the group of patients older than 10 years old was, respectively, 7.8 h/day and 5.9 h/day. However, this difference was not statistically significant. 

When defining daytime as 8 a.m.–8 p.m., the mean number of hours of use was 1 h per day versus 5.7 h of use at night-time, defined as by 8 pm–8 am, which means that for 15% of the time the headgear was used during the day while for the remaining 85% of the time it was used at night. This difference was statistically significant (Table 3). 

## 4. Discussion

For the correction of Class II malocclusions, various devices are available, some of which are compliance-dependent, such as removable functional appliances, and others are not, such as the Pendulum or the Carriere Motion [14,15,16]. Class II malocclusion can also be treated by the use of a bone-borne intraoral distalizing appliance that has the advantage of overcoming the typical need for patient compliance with extraoral appliances [15,17]. However, in this study, the focus was on the use of extraoral traction, which, although highly compliance-dependent, can yield excellent results if worn [18]. The existing literature highlights how monitoring the use of headgear could improve collaboration, but there is still a lack of detailed studies measuring this effect quantitatively. Thus, the primary objective of this study was to carefully assess how well patients work with headgear using objective tools for evaluation. Additionally, we wanted to explore how knowing that they are being monitored might affect how often they use these devices. That is why it was investigated how to measure and influence collaboration in this context. Objective methods are currently available to assess the patient’s level of cooperation in wearing headgear; reference is made to modern sensors such as Smartgear Compliance Monitoring Device, that is sensitive to changes in temperature and also applied force. Detecting both of these parameters allows minimizing the chances of error, enabling the device to record more accurately when the extraoral traction is worn and when it is removed.

With the Smartgear device, it appears that, over the course of three months, the study group used the equipment for an average of 7.3 h per day, while the control group averaged 6.2 h, indicating a difference of over an hour. Assessing the average daily collaboration in terms of hours for all individual patients throughout the 3-month observation period, it can be stated that almost none were able to match or even surpass the clinician-prescribed hours of 13 h per day. A total average daily compliance of 6.7 h was highlighted, which appears to be low, as the rate of cooperation was approximately 46.15%. This pattern has been highlighted by numerous studies; however, the reason is not yet entirely clear [9]. Nevertheless, it could be attributed to the discomfort of the appliance, coupled with social effects such as embarrassment. 

The study conducted by Brandão et al. also sought to address the question of whether the awareness of being monitored throughout the treatment period could positively impact the outcome. Initially, no information was provided in the patient group regarding the monitoring of headgear use. In this context, the average of actual hours of daily use compared to clinician requests was 56.7%. Subsequently, the same group of patients was informed about monitoring and this awareness led to an increase in the rate of cooperation to 62.7% [10]. In the current study, two groups of patients were randomly selected: a control group, not informed of monitoring, and a study group, informed of monitoring. The statistical analysis of average daily compliance between the two groups revealed a difference of 1.1—corresponding to just over one hour. In detail, the study group showed an average daily cooperation of 7.3 h per day during the three months of observation, while the control group recorded 6.2 h. Thus, a modest increase was observed in the group that was aware of monitoring, a result in line with what has been reported in other studies in the literature; however, this difference was not found to be statistically significant. Based on observation of the mean alone, there emerges a slight propensity to use the device more knowing that the latter monitors the time of use. 

Evaluating in terms of hours the average daily collaboration of all individual patients throughout the 3-month observation time (regardless of the group they belonged to), it is possible to state that almost no one (only two patients) was able to match or even exceed the clinician’s prescription hours corresponding to 13 h/day. This trend agrees with other studies in the literature such as that of Huanca et al., which states that the cooperation of patients with headgear turns out to be poor [19], and it also explains why a good share of clinicians are disinclined to use headgear as described by the article of Tüfekçi et al. [20]. Many studies have sought to understand the factors that influence compliance [8,21,22]. According to Mehra et al. study, the patient’s desire for orthodontic treatment should be assessed at the beginning of the treatment process as it is one of the most important predictive factors for cooperation [23]. Now, the issue arises when using headgear, especially for skeletal effects, which must be done at an early age when the patient may lack an awareness of their own condition. And it is here that the relationship with one’s parents and the tendency to listen to them could lead to a different outcome. 

Collaboration pattern analysis performed through the Smartgear Compliance Control System software allows us to understand how patients used headgear inconsistently during the treatment period. As highlighted in the study conducted by Almuzian et al., discontinuous use results in the intermittent application of forces, which can lead to biomechanical disadvantages and an increased risk of recurrence [24]. The study revealed that patients exhibiting lower adherence levels displayed distinct patterns, characterized by intermittent periods of complete non-usage of the orthodontic device, interspersed with phases of daily utilization. Notably, instances of single-day forgetfulness were infrequent during the observation period. This observation is supported by the literature, as evidenced in the study conducted by Huanca et al., which reported that, during an observation period of 8 months, the patients considered used headgear for a total of 5.8 months, with a period of non-use of the orthodontic appliance of 2.6 months, corresponding to 30% of the time [19]. Also, in the same study, there was evidence of a higher frequency of use during the night than during the day: this same trend was observed in this group of patients. Indeed, the current investigation demonstrated that patients, on average, wore their appliance predominantly during night-time hours, constituting 85% of the total usage time, spanning the period from 8 p.m. to 8 a.m. More precisely, the mean daily utilization over the three-month observation period amounted to a mere 1 h during daytime hours, significantly contrasting with the 5.7 h of night-time usage, validated by a statistically significant difference. It must be considered that daytime cooperation by patients is more difficult because of the now greater activities that tend to fill the patient’s day: sports activities, extracurricular activities, and social activities. All this necessarily limits the amount of time our patient can devote to using the equipment without any attached risks or without feeling uncomfortable because of the particularly bulky and unsightly device. 

Another question sought to be answered was whether there iwass a statistically significant difference in mean monthly compliance between the first month and the last month of treatment. Arreghini et al. also tried to answer the same question, and found that the average compliance during the first 5 months of treatment was constant while thereafter it tended to decrease, although not statistically significantly [12]. In the current study, what was shown was fairly good compliance in the first month (7.6 h/day) and slightly lower compliance in the last month (6.2 h/day). It can be said, therefore, that there is indeed greater compliance in the first month than in the last, albeit small and not statistically significant, which is likely due to the initial enthusiasm of the patient. Through studies, it has been observed that compliance during prolonged orthodontic treatment may worsen. One potential explanation for this discovery revolves around the concept of “patient burnout.” As orthodontic treatments often address many esthetic issues within the initial months, patients may begin to feel that no further action is necessary. However, orthodontists understand that achieving the desired occlusal results requires additional treatment time. This disconnect between patient perceptions and orthodontists’ expectations could lead to a decline in patient compliance [25,26].

Also, in the study by Arreghini et al., it was investigated whether there was a difference, in terms of treatment adherence, between female and male patients and between patients of different ages. What emerged was that there was no difference between the two genders, while the same could not be said in relation to age: young patients (6–8 years) were significantly more cooperative than adolescents (12–15 years) [12]. Considering the patients in this study, slightly higher compliance was shown by males than females. This result aligns with findings from the study by Brandão et al. [10]; however, in the literature, several authors have noted that girls tend to be more cooperative than boys when it comes to wearing headgear [21,27,28,29].

Regarding age differences, a pattern similar to the one described in Arreghini et al.’s article [12] was observed. There was a greater tendency for collaboration among patients aged 10 or younger compared to those who were older. This pattern holds true both for the entire sample examined and when considering the individual control and study groups.

Specifically, the average daily compliance for patients aged 10 or younger was 7.8 h per day, while those older than 10 years reported an average of 5.9 h per day. Within the study group, children aged 10 or younger showed an average daily compliance of 7.6 h, while their counterparts who were older than 10 years old report 7.1 h. In the control group, patients aged 10 or younger exhibited an average daily cooperation of 7.9 h, whereas those older than 10 years old had an average of only 4.2 h of daily cooperation. Although the difference was not statistically significant, it is worth noting that younger children tended to use the device for almost 2 h more per day on average than older children. This result is consistent with the existing literature, which suggests that younger patients tend to exhibit a higher level of compliance [3,6,30,31]. One potential reason behind this discovery could be that younger children, before adolescence, may tend to be more open to and compliant with parental guidance, making them more likely to follow instructions compared to adolescents.

Unfortunately, primarily due to the considerably small sample size, the study did not yield conclusive results. Nevertheless, it is believed that the use of the monitoring device employed holds significant promise. For years, clinicians using headgear have frequently encountered impasses, unsure of the underlying issues. This was compounded by reports from patients and, primarily, their parents, who claimed regular appliance wear even when it was not the case.

The study aimed to better understand whether the awareness of being monitored could positively influence compliance with therapy. Although the sample size was small, the findings align with the existing literature, indicating that awareness alone does not impact compliance. Significant variation among patients was observed, and the literature confirms that extraoral traction, if used appropriately, remains an excellent approach for correcting Class II malocclusion with maxillary prognathism before considering more complex and invasive treatments.

The sensor used in this study provides precise measurements of patient cooperation over a few months. This early assessment allows clinicians to make informed decisions about whether to continue with the current therapy or explore alternative methods. By acquiring objective data on patient adherence, the device aids in tailoring the treatment plan to the individual’s needs and avoids the pitfalls of prolonged, ineffective treatment, which can lead to patient fatigue and frustration.

Using the sensor enables orthodontists to gain valuable insights into the patient’s cooperation level and personality early on. This helps determine whether to persist with the current approach or to consider other strategies, which, while potentially more invasive and costly (e.g., skeletal anchorage devices), might be necessary if initial cooperation is inadequate. Understanding patient cooperation early in the treatment process prevents wasted time and effort on methods that may ultimately fail, thereby improving both clinician and patient satisfaction.

We conclude by stating that this study had a sample size of 22 patients, which represents the major limitation of the present work, along with a relatively short observation period of only 3 months. This led to results consistent with the literature but without adequate statistical significance. Therefore, further studies are needed to increase the number of patients included in the sample and to extend the observation period, as these changes could potentially alter the obtained results. For example, it might be beneficial in the future to extend the observation period and exclude the initial weeks of equipment usage, as patients need time to acclimatize to its use, especially considering its bulkiness, which may not lead to immediate adaptation. Another limitation stems from the clinician’s awareness of the patient’s membership in a particular group, potentially influencing their behavior. However, it should be noted that the device used to assess collaboration is quite identifiable to the clinician. One possible solution could be to develop a detection device that is indistinguishable even to the clinicians themselves, thus enabling the implementation of a double-blind study.

## 5. Conclusions

Considering the small sample size examined, we can conclude as follows:

- There was no significant difference in compliance between patients aware of the monitoring and those unaware, with the study group showing only an additional 1.1 h per day of compliance.
- Patients’ compliance tended to be slightly higher in the first month and then decreasing. Overall, there did not appear to be a statistically significant between months of treatment.
- The average daily use was 6.7 h, which is notably below the 13 h daily recommendation, indicating only 46.15% adherence.
- Patients predominantly used the headgear at night (85%) rather than during the day (15%), with this difference being statistically significant.
- Males and females showed no statistically significant differences in cooperation.
- Children aged 10 years or younger showed slightly better adherence to therapy compared to older children, though this result was not statistically significant.
- Despite the lack of significant differences, the sensitive device remains a valuable tool for improving patient communication and providing objective data on cooperation. It is also useful for assessing early in the treatment whether to proceed with the headgear or consider an alternative approach.

## Figures and Tables

**Table 1:** Average monthly compliance (h/die) for each group of patients under study.

Group	Month 1	Month 2	Month 3
Study group	6.9	6.5	6.7
Control group	8.3	6.3	5.8

**Table 2:** Average daily compliance (h/day) for each month of the patients under study.

PATIENT	Month 1	Month 2	Month 3
1	9.3	10.6	10.0
2	8.2	7.9	6.5
3	10.1	3.1	0.0
4	16.2	15.5	14.6
5	1.5	1.1	1.1
6	1.7	1.4	3.7
7	13.3	13.0	15.7
8	12.1	11.5	10.9
9	8.0	7.8	6.1
10	2.7	3.5	3.2
11	7.4	5.5	7.1
12	7.0	5.7	5.6
13	12.9	11.9	11.9
14	2.4	4.5	6.3
15	7.3	5.5	2.5
16	9.9	5.0	5.7
17	7.8	1.3	0.6
18	8.8	9.2	9.5
19	3.4	5.2	5.7
20	1.4	0.3	0.6
21	8.2	5.8	2.2
22	7.6	5.6	7.5

**Table 3:** Compliance with headgear and statistical significance ^a^.

Category	N	Mean Compliance (Hours per Day)	SD	MIN	MAX	MEDIAN	SE	Test	*p*-Value
Awareness monitoring									
Informed	11	7.3	4.3	1.2	15.4	6.7	1.3	Student’s t Wilcoxon–Mann–Whitney	0.54 0.65
Not informed	11	6.2	3.7	0.7	12.2	5.4	1.1		
Observation period									
Month 1	22	7.6	4.1	1.4	16.2	7.9	0.9	ANOVA Kruskal–Wallis	0.51 0.32
Month 2	22	6.4	4.1	0.3	15.5	5.6	0.9		
Month 3	22	6.2	4.5	0	15.7	5.9	0.9		
First month vs. last month									
Month 1 (first month)	22	7.6	4.1	1.4	16.2	7.9	0.9	Student’s t Wilcoxon–Mann–Whitney	0.30 0.16
Month 3 (last month)	22	6.2	4.5	0	15.7	5.9	0.9		
Gender									
Girl	9	6.2	3.7	0.7	12.2	6.9	1.2	Student’s t Wilcoxon–Mann–Whitney	0.60 1.00
Boy	13	7.1	4.2	2.3	15.4	6.1	1.2		
Age									
≤10	10	7.8	2.9	3.2	12.2	7.2	0.9	Student’s t Wilcoxon–Mann–Whitney	0.26 0.14
>10	12	5.9	4.6	0.7	15.4	4.9	1.3		
Use during the day									
8 a.m.–8 p.m.	1980	1	1.9	0	11.8	0	0.04	Student’s t	0 *
8 p.m.–8 a.m.	1980	5.7	4.3	0	12	7.5	0.10		

^a^ N indicates sample size; SD, standard deviation; MIN, minimum; MAX, maximum; SE, standard error; * *p*-value < 0.05 (statistically significant).

## Data Availability

The raw data supporting the conclusions of this article will be made available by the authors on request.
